# ImmunoRatio: a publicly available web application for quantitative image analysis of estrogen receptor (ER), progesterone receptor (PR), and Ki-67

**DOI:** 10.1186/bcr2615

**Published:** 2010-07-27

**Authors:** Vilppu J Tuominen, Sanna Ruotoistenmäki, Arttu Viitanen, Mervi Jumppanen, Jorma Isola

**Affiliations:** 1 Institute of Medical Technology, University of Tampere, Biokatu 6, 33014 Tampere, Finland; 2 Department of Pathology, Seinäjoki Central Hospital, Hanneksenrinne 7, 60220 Seinäjoki, Finland

## Abstract

**Introduction:**

Accurate assessment of estrogen receptor (ER), progesterone receptor (PR), and Ki-67 is essential in the histopathologic diagnostics of breast cancer. Commercially available image analysis systems are usually bundled with dedicated analysis hardware and, to our knowledge, no easily installable, free software for immunostained slide scoring has been described. In this study, we describe a free, Internet-based web application for quantitative image analysis of ER, PR, and Ki-67 immunohistochemistry in breast cancer tissue sections.

**Methods:**

The application, named ImmunoRatio, calculates the percentage of positively stained nuclear area (labeling index) by using a color deconvolution algorithm for separating the staining components (diaminobenzidine and hematoxylin) and adaptive thresholding for nuclear area segmentation. ImmunoRatio was calibrated using cell counts defined visually as the gold standard (training set, *n *= 50). Validation was done using a separate set of 50 ER, PR, and Ki-67 stained slides (test set, *n *= 50). In addition, Ki-67 labeling indexes determined by ImmunoRatio were studied for their prognostic value in a retrospective cohort of 123 breast cancer patients.

**Results:**

The labeling indexes by calibrated ImmunoRatio analyses correlated well with those defined visually in the test set (correlation coefficient *r *= 0.98). Using the median Ki-67 labeling index (20%) as a cutoff, a hazard ratio of 2.2 was obtained in the survival analysis (*n *= 123, *P *= 0.01). ImmunoRatio was shown to adapt to various staining protocols, microscope setups, digital camera models, and image acquisition settings. The application can be used directly with web browsers running on modern operating systems (e.g., Microsoft Windows, Linux distributions, and Mac OS). No software downloads or installations are required. ImmunoRatio is open source software, and the web application is publicly accessible on our website.

**Conclusions:**

We anticipate that free web applications, such as ImmunoRatio, will make the quantitative image analysis of ER, PR, and Ki-67 easy and straightforward in the diagnostic assessment of breast cancer specimens.

## Introduction

Immunohistochemical staining of the estrogen receptor (ER), progesterone receptor (PR), and proliferation antigen Ki-67 are routinely used in the diagnostic assessment of breast cancer. Positive ER status of a tumor is considered necessary for patients to be eligible for post-surgical hormonal therapies. ER and PR assays are based on immunohistochemistry performed on formalin-fixed and paraffin-embedded tumor tissue blocks [1]. Although the analytical quality of ER and PR assays has been debated for decades, recent results of interlaboratory quality assurance studies provide convincing evidence for the high reproducibility of these laboratory staining procedures [2,3]. The tumor cell proliferation antigen level, as defined by Ki-67 immunostaining, is an auxiliary tool for defining patient prognosis. Patients with rapidly proliferating tumors are predicted to endure poorer outcomes than patients with tumors exhibiting low proliferation [4]. Meta-analyses confirming the role of Ki-67 as a prognostic factor have included more than 15,000 patients [5].

Common practice in pathology laboratories is to score ER-, PR-, and Ki-67-stained slides visually (also termed manually) using light microscopy at medium power magnification (10× or 20× objectives). For ER and PR evaluation, a tumor is scored as negative or positive or, as is currently recommended, by evaluating the percentage of positively stained tumor cell nuclei [6]. A threshold of 10% total stained tumor cells is commonly used as a cut-off for defining positive ER and PR status. A combination of the stained cell percentage and the staining intensity is applied in histoscore and Allred-score methods [7,8]. Whichever scoring method is used, it is well known that microscopic evaluation of ER- and PR-stained slides is subjective and can lead to significant inter-observer variability. For example, in an extensive inter-laboratory study of 172 pathologists, 24% of ER-positive slides were interpreted as falsely negative [9]. Interpretation of Ki-67 staining can be even more difficult, mainly owing to the lack of uniformly accepted cut-off points for defining low- and high-risk patient groups. In most of the published studies included in the meta-analyses, Ki-67 has been evaluated in a single center or by one or very few observers, thereby failing to address the problem of possible inter-observer variability [4,5]. The magnitude of inter-observer variability for Ki-67 scoring is largely unknown, but there is no reason to believe that it would be less than that of ER and PR.

In clinical practice, ER-, PR- and Ki-67-stained slides are interpreted by a pathologist. Careful estimation of the percentage of positively stained cells (labeling index) is not only prone to inter-observer variation, but is also tedious and time-consuming. To overcome this, various digital image analysis methods have been described [10,11]. The principles behind quantitative immunohistochemistry analyses are based on differentiation of the staining components by using, for example, the color deconvolution algorithm [12]. The color deconvolution algorithm detects and separates multiple stains by analyzing their absorption spectra and relative contributions to areas containing two or more overlapping stains. Although the optical density of the immunoreaction product (brown diaminobenzidine (DAB) precipitate) may not accurately reflect the abundance of the antigen (ER, PR, or Ki-67 protein), systems discriminating between negatively and positively stained cells have turned out to be useful [13]. Unfortunately, the image analysis software described in the literature is seldom released for public use. Likewise, the commercially available software is usually proprietary and/or bundled with dedicated analysis equipment or virtual microscopy scanners, making it difficult to compare them [14].

In order to become widely accepted and utilized by pathologists, a digital image analysis system should be easily accessible, not require dedicated equipment or software installation, and be compatible with existing microscope setups. For this purpose, we developed an image analysis application, named ImmunoRatio, which is accessed and used within a web browser. ImmunoRatio supports all modern web browsers and operating systems, requiring no software installation. The application segments immunostained and hematoxylin-stained cellular areas from the user-submitted image and calculates the labeling index (percent of DAB-stained area out of the total nuclear area). ImmunoRatio is free, open source, and publicly available on our research group website [15].

## Materials and methods

### Immunohistochemistry

Formalin-fixed and paraffin-embedded tissue sections from invasive breast cancers were derived from the archive of the Department of Pathology, Seinäjoki Central Hospital. The study has been approved by the Scientific Committee of Seinäjoki Central Hospital, Finland. According to the Finnish national ethics committee regulations, informed consent was not considered necessary for this study. Immunohistochemical stainings of ER, PR, and Ki-67 tissue sections followed the recommended staining protocols [3]. The slides were stained using the BondMax staining robot (Leica Microsystems, Wetzlar, Germany). In brief, ER was detected using monoclonal antibody 6F11 (diluted 1:300, Leica Biosystems, Newcastle, UK), PR was detected using monoclonal antibody PgR636 (diluted 1:600, Leica Biosystems, Newcastle, UK), and Ki-67 was detected using monoclonal antibody MIB-1 (diluted 1:100, Dako, Carpinteria, CA, USA). Antigen retrieval was performed in Tris-EDTA buffer (pH 9, 100°C for 40 minutes). Bound antibodies were visualized using Bond Refine Detection kit (Leica Biosystems, Newcastle, UK). Immunoreaction was intensified using 0.5% copper sulfate (5 minutes). Hematoxylin counterstaining (1 minutes in ChemMate diluted 1:6, Dako, Carpinteria, CA, USA) was performed using PBS as bluing reagent. The samples were cleared with ethanol and xylene and mounted using standard procedures.

### Prognostic validation

Samples from 123 primary breast cancer patients were derived from the archives of the Department of Pathology at Tampere University Hospital, with the permission from National Supervisory Authority for Welfare and Health (Köninki *et al*.: Analysis of PIK3CA mutations and protein expression in breast cancer, submitted). Survival rates of all patients were calculated by the method of Kaplan and Meier. Data on breast cancer-specific mortality was obtained from Finnish Cancer Registry. Up to 20-year follow-up was available for this patient cohort (cancers diagnosed between 1988 and 1992). The immunohistochemical staining for Ki-67 was carried out as described above, except that PowerVision+ kit (ImmunoVision, Springdale, AZ, USA) was used for antibody detection and LabVision Autostainer (LabVision, Fremont, CA, USA) for staining automation. Informed consent in very old retrospective patient cohorts was deemed unnecessary, because the study was approved by the local hospital ethics committee and the National Supervisory Authority for Welfare Health.

### Image acquisition

Digital images were captured using a Leica DM3000 microscope (Leica Microsystems, Wetzlar, Germany) equipped with 10×, 20×, and 40× objective lenses, a 1× phototube, and a Scion CFW-1612C digital color camera (Scion Corporation, Frederick, MD, USA; 24-bit color depth; resolution 1,600 × 1,200 pixels, pixel size 4.40 μm). The images were stored using an uncompressed image file format (bitmap). For every imaging session, an image from empty slide background area was acquired (blankfield image), which was used to correct image color balance and uneven illumination. Optimal image brightness and contrast were determined by using the Camera Adjustment Wizard, which was developed as an incorporated function of ImmunoRatio. The Camera Adjustment Wizard measures the brightness of the blankfield image and performs a contrast analysis using an image containing hematoxylin-stained cells. An optimal brightness (mean gray intensity) of the blankfield image is considered to be in the range of 200 to 250 (available range 0 to 255, black being 0). The contrast analysis segments the hematoxylin-stained cells (foreground) and analyzes their mean gray intensity, which is then divided by the background mean gray intensity. The contrast is considered to be optimal if the foreground mean gray intensity is 50 to 80% of the background mean gray intensity.

### Software development

**Figure 1:**
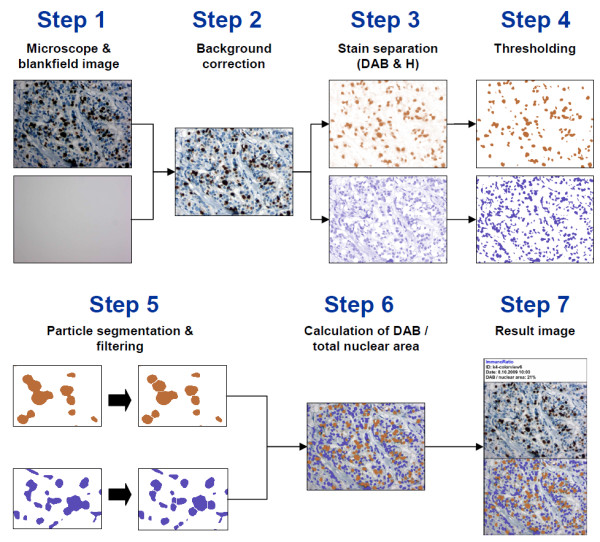
**A flowchart outlining the ImmunoRatio analysis algorithm**. **Step 1: **A RGB color microscope image, an optional blankfield correction image, and thresholding adjustment parameters are received as an input. **Step 2: **The blankfield image is used to correct uneven illumination and color balance. If a blankfield image is not available, background subtraction is carried out using the Rolling ball algorithm [19]. **Step 3: **The Color Deconvolution plugin [20] is used to separate the stains into two eight-bit component images: diaminobenzidine (DAB) and hematoxylin (H). **Step 4: **The components are processed with a mean filter and binarized using adaptive IsoData thresholding [21]. Component-specific threshold adjustments are applied if defined via input parameters. **Step 5: **The components are processed with a median filter to smooth the thresholding result. Nucleus segmentation is performed on both components by using the Watershed algorithm [22] and small particles are discarded based on their size. For the H component, thin (fibroblastic) cells are identified and discarded using non-round particle removal. **Step 6: **The H and DAB components are overlaid on the source image. The percentage of DAB-stained nuclear area out of the total nuclear area (the labeling index) is calculated. An (optional) external calibration function is used to correct the ratio percentage. **Step 7: **The result image consisting of image identification string, the analysis date, the result labeling index, the original image, and a pseudo-colored image showing the staining components is created. A more detailed algorithm flowchart is available on our research group website [15].

ImmunoRatio was first developed as a plugin for the ImageJ image analysis software (1.42 m) [16] using the Java programming language [17]. In addition to built-in ImageJ functions, the ImmunoRatio analysis algorithm uses the Calculator Plus plugin [18] for blankfield correction, the Rolling Ball algorithm [19] for background subtraction, the Color Deconvolution plugin [20] for DAB and hematoxylin stain separation, the IsoData algorithm [21] for adaptive thresholding, and the Watershed algorithm [22] for nucleus segmentation. The analysis algorithm steps are outlined in Figure 1. A more detailed algorithm flowchart is available on our website [15]. The ImmunoRatio plugin was embedded into a Java servlet-based web application. The web application was developed using Google Web Toolkit (1.7.0) [23], Apache Commons FileUpload package (1.2.1) [24], Apache Commons IO library (1.4) [25], Laboratory for Optical and Computational Instrumentation Bio-Formats package (4.1) [26], and Apache Tomcat servlet container (6.0) [27].

### Software calibration

From a pool of 100 immunohistochemically stained slides, 50 were selected to be included in the training set (25 stained for Ki-67, 13 for PR, and 12 for ER). The labeling indexes (percentage of positively stained nuclei by visual assessment) were evenly distributed, ranging from 0 to 100%. From each training set slide, one image representative for invasive carcinoma was acquired. Each acquired image was analyzed visually by counting positively and negatively stained carcinoma cells on a computer screen (a minimum of 500 cells total per image). The percent of DAB-stained nuclei out of the total nuclei (DAB- and hematoxylin-stained) was calculated as the labeling index. This result was used as the gold standard for ImmunoRatio calibration. The images were then analyzed using non-calibrated ImmunoRatio, and the results were compared with visual counting in a scatter plot. Owing to the non-linear relation, a third degree polynomial was fitted to the data. ImmunoRatio was then calibrated by embedding the fitted polynomial as a correction function into the analysis algorithm. To validate the calibration and demonstrate the accuracy of the analysis, the remaining 50 samples (25 stained for Ki-67, 13 for PR, and 12 for ER) were used as a test set, which was analyzed using calibrated ImmunoRatio. In the final step of the validation, the minimum number of images needed to be averaged from a typical tumor sample (diameter 1 to 2 cm) was defined. From 10 samples, 12 images per sample representing central and peripheral tumor areas were acquired using 20× objective.

### Software testing

ImmunoRatio was initially developed and calibrated using ER-, PR-, and Ki-67-stained slides, which were considered optimal by an external quality assurance program [3]. To simulate interlaboratory variability in staining results, the effect of suboptimal primary antibody (Ki-67 MIB-1) dilution and hematoxylin counterstaining intensity was studied. The robustness of ImmunoRatio to variations in image acquisition settings was examined by comparing the optical resolutions provided by 10×, 20×, and 40× microscope objectives, and by comparing the analysis results obtained with six microscope cameras: Scion CFW-1612C (Scion Corporation, Frederick, MD, USA), Altra 20 (Olympus Corporation, Tokyo, Japan), ColorView II (Olympus Corporation, Tokyo, Japan), Leica DFC290 HD (Leica Microsystems, Wetzlar, Germany), Mightex 3MP Color CMOS (Mightex Systems, Pleasanton, CA, USA), and Nikon DS-Fi1 (Nikon Corporation, Tokyo, Japan). The primary output file format used in the acquisition was uncompressed (i.e., lossless). Images were also acquired using JPEG file format (quality factors 10, 20, 40, 60, 80, and 100) to study the suitability of lossy compression for ImmunoRatio analysis. In addition, for each camera, the average diameter (pixels per μm) of a hematoxylin-stained nucleus was measured. Linear regression was used to fit a first degree polynomial to the data and the polynomial was then embedded into the Scale Finder function of ImmunoRatio. The Scale Finder assists the user in determining a rough scale estimate for the microscope setup, if not known prior to analysis.

## Results

### ImmunoRatio software

**Figure 2:**
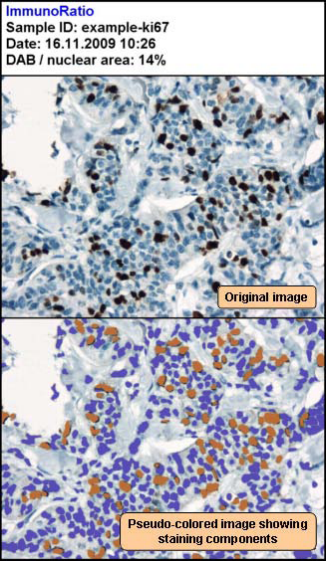
**An example result of a Ki-67-stained image processed with ImmunoRatio**. The result image includes a sample identifier, the analysis date, the labeling index (percentage of positively stained nuclear area), the original image, and a pseudo-colored image showing the segmented staining components.

We developed the ImmunoRatio image analysis software, which segments the DAB- and hematoxylin-stained nuclei areas from a microscope image, calculates the labeling index (percent of DAB-stained area out of the total nuclear area), and generates a pseudo-colored result image matching the segmentation. An example analysis output of a Ki-67 image is shown in Figure 2. We first implemented ImmunoRatio as an open source ImageJ plugin, which provides a graphical user interface, as well as the possibility to use it with ImageJ macro language. Multiple images from the same specimen can be analyzed at once, resulting in a montage containing all of the analyzed images. The plugin version enables a direct link to image capture either by using the driver plugins provided by the camera vendors or via the open TWAIN protocol [28]. An open source version of the plugin is available for free download [29].

**Figure 3:**
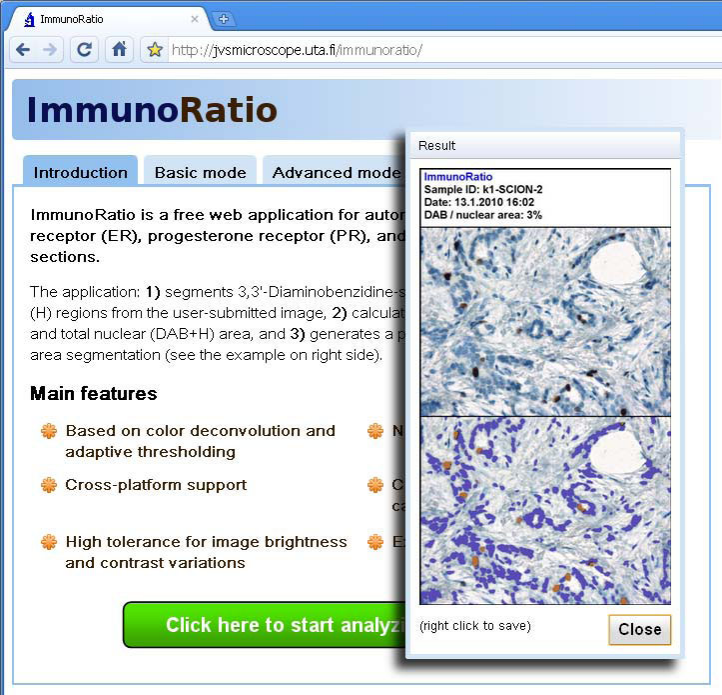
**A screenshot of the ImmunoRatio web application**. The Result window is shown as an insert in the right panel. The application is publicly available on our jvsmicroscope.uta.fi website, where users can analyze their images freely.

Based on the plugin described above, we developed a publicly available ImmunoRatio web application (see screenshot in Figure 3). The web application resides in a remote server and is accessed over the Internet with a web browser, without any software downloads or installations. It supports all modern web browsers (e.g., Windows Internet Explorer, Mozilla Firefox, Safari, and Google Chrome) and all operating systems (e.g., Microsoft Windows, Linux distributions, and Mac OS). The main features of the ImmunoRatio web application are summarized in Table 1. The analysis is based on the color deconvolution [12] for stain separation and adaptive IsoData algorithm [21] for thresholding. The analysis can be made either to the whole image or to an interactively defined region of interest (ROI). The analysis adapts to various combinations of microscope objective lenses, phototubes, and camera resolutions by using either an exact or an estimated image scale (pixels per μm). The estimation can be performed using the Scale Finder function. ImmunoRatio supports most existing camera models and their output images, including JPEG, JPEG2000, TIFF, BMP, and PNG. Optimal camera brightness and contrast settings can be defined using the assistance of the Camera Adjustment Wizard. Users can calibrate the software with their own visually determined labeling index data and derive a suitable result correction equation (a third degree polynomial). Users can also fine-adjust the hematoxylin- and DAB-thresholding parameters. For demonstrational analyses, ImmunoRatio offers an introductory basic mode, which has a simplified user interface with minimal required functionality. ImmunoRatio web application is freely accessible on our research group website [15].

**Table 1:** Main features of ImmunoRatio web application

Feature	Description
**Analytical principle**	Analyzes immunostained slides (ER, PR, Ki-67) using color deconvolution [12] for stain separation and adaptive IsoData algorithm [21] for thresholding. Users can analyze either the whole image or a region of interest.
**Hardware and software requirements**	Runs within the web browser, requiring no additional program or plugin installations. Is compatible with all modern web browsers and operating systems.
**Compatibility with different microscope setups**	Adapts to various combinations of microscope objective lenses, phototubes, and camera resolutions. Supports most existing camera models and image formats (JPEG, JPEG2000, TIFF, BMP, PNG). Users can define optimal camera brightness and contrast settings with the Camera Adjustment Wizard.
**Calibration**	Users can calibrate the application to match with their own visual cell counting data.
**Usage modes**	Includes a basic mode for introductory analyses and a full-featured mode.

ER, estrogen receptor; PR, progesterone receptor.

### Calibration of ImmunoRatio

**Figure 4:**
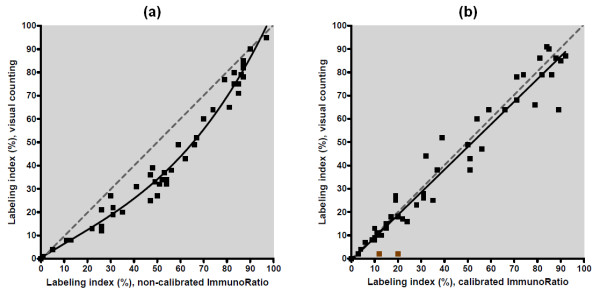
**Scatter plots comparing labeling indexes defined by visual cell counting, non-calibrated ImmunoRatio, and calibrated ImmunoRatio**. **(a) **The calibration was made using a training set of 50 samples, of which 25 were stained for Ki-67, 13 for progesterone receptor (PR), and 12 for estrogen receptor (ER). To achieve linear relation (dotted line), a correction function was defined by fitting a third degree polynomial (solid black line) to the training set. **(b) **The calibration was validated by using a separate test set of 50 samples (25 stained for Ki-67, 13 for PR, and 12 for ER). The validation test set included two outliers (marked as brown).

Although non-calibrated ImmunoRatio correlated well with visual counting of DAB- and hematoxylin-stained cell nuclei (*r *= 0.97), the results showed an obvious non-linear relation (Figure 4a). Due to this non-linearity, a third degree polynomial was fitted to the data and used as a correction function to calibrate ImmunoRatio. The analysis of the separate test set with calibrated ImmunoRatio had a strong linear relation with visual cell counting, showing a near-perfect correlation (*r *= 0.98; Figure 4b). The test set included two outlier observations, which were detected by visually inspecting the pseudo-color result images. The first outlier had weak DAB-staining intensity, making interpretation based on visual counting difficult. The second outlier had too low image contrast as demonstrated by using the Camera Adjustment Wizard.

**Figure 5:**
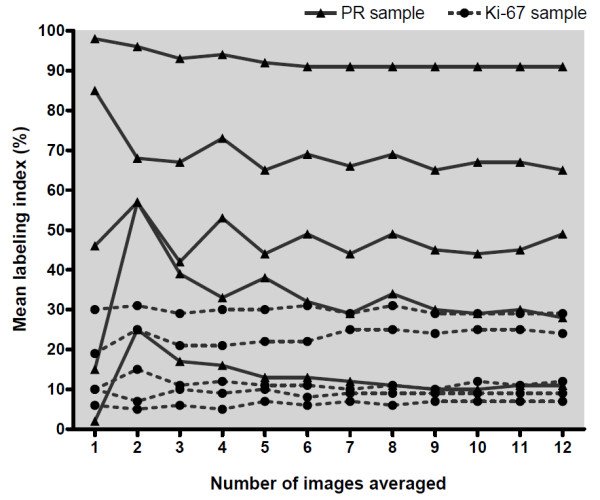
**The mean labeling index of ImmunoRatio analysis as a function of the number of images included in the averaged result**. Five samples stained for progesteron receptor (PR) and five for Ki-67 were tested.

In the final step of the validation process, we defined the minimum number of images needed to be captured and analyzed in order to obtain a representative result for the stained breast tumor slides. Using 20× microscope objective, a sufficient number of images per sample for accurate ImmunoRatio analysis was determined to be three (Figure 5). Averaging data from a higher number of images was found to have a minimal impact on the mean labeling index.

### The effect of variability in staining and image acquisition settings

**Figure 6:**
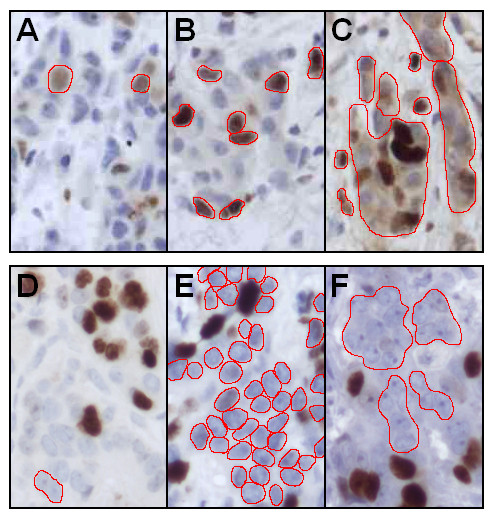
**The importance of optimal immunostaining conditions on the accuracy of ImmunoRatio analysis**. The red lines outline the nuclei and highlight the segmentation of **(a to c) **brown and **(d to f) **blue staining components. **(a) **Overly dilute primary antibody concentration (Ki-67 MIB-1, 1:400) causes inadequate brown segmentation. **(b) **Optimal antibody dilution (1:100). **(c) **Overly strong antibody concentration (1:25) results in excessive cytoplasmic staining and brown segmentation. **(d) **Overly dilute hematoxylin staining causes inadequate blue segmentation. **(e) **Optimal hematoxylin dilution. **(f) **Overly strong hematoxylin causes excessive cytoplasmic staining and blue segmentation.

The compatibility of ImmunoRatio with variable staining and image acquisition settings is summarized in Table 2. An optimally titrated primary antibody (1:100 for MIB-1 Ki-67) resulted in the best match with visual cell counting. ImmunoRatio tolerated substantial deviations in the antibody dilutions well. A usable antibody dilution was 1:50 to 1:200, because a four-times more diluted antibody (1:400) resulted in labeling indexes that were too low, whereas using very concentrated antibody (1:25) led to cytoplasmic background staining and labeling indexes that were too high. Optimal hematoxylin counterstaining was found to be important. Weak counterstaining caused the nuclear segmentation to fail, whereas overly concentrated counterstaining led to false segmentation of the cytoplasmic structures. Sample images with optimal and non-optimal primary antibody and hematoxylin counterstaining are presented in Figure 6.

**Table 2:** The compatibility of ImmunoRatio with variable staining and image acquisition settings

Immunostaining/image acquisition feature	Compatibility with ImmunoRatio	Comments
**Primary antibody dilution** **(defined for MIB-1 Ki-67)**		

too dilute (1:400)	**+**	too low labeling index (Figure 6a)
optimal (1:100)	**+ + +**	best match with visual counting (Figure 6b)
too strong (1:25)	**+**	cytoplasmic background causing overly high labeling indexes (Figure 6c)

**Hematoxylin counterstaining**		

weak	**-**	insufficient nuclear segmentation (Figure 6d)
optimal	**+ + +**	best match with visual counting (Figure 6e)
strong	**+**	false segmentation of cytoplasmic structures (Figure 6f)

**Microscope objective magnification** **(using 1× phototube)**		

10×	**+**	non-carcinomatous cells often included*
20×	**+ + +**	for accurate result, an average of three images per sample is recommended
40×	**+ +**	for accurate result, averaging several images per sample is recommended

**Image brightness**		

underexposed (too dim)	**+**	mean gray intensity of the blankfield image <200
in optimal range	**+ + +**	as guided by the Camera Adjustment Wizard of ImmunoRatio
overexposed (too bright)	**-**	mean gray intensity of the blankfield image >250

**Image contrast**		

too low	**-**	foreground mean gray intensity over 85% of the background mean gray intensity
in optimal range	**+ + +**	as guided by the Camera Adjustment Wizard of ImmunoRatio
too high	**+**	foreground mean gray intensity under 50% of the background mean gray intensity

**Image compression**		

uncompressed (lossless)	**+ +**	slow network transmission (slower overall analysis time)
JPEG, quality factor 50 to 100 (lossy)	**+ + +**	optimal for ImmunoRatio
JPEG, quality factor <50 (lossy)	**-**	visible image artifacts

+ + + = optimally compatible, + + = compatible, + = compatible with possible chance of analytical errors, - = not compatible with ImmunoRatio.

* Region of interest function can be used to exclude unwanted tissue areas.

Owing to the Scale Finder function, the results of images acquired using 10×, 20×, and 40× objective lenses (with a 1× phototube) were highly similar (data not shown). The 20× objective was deemed to be optimal, requiring at least three images per sample to be averaged. The same results could be achieved by using the 40× objective, but more images per sample needed to be averaged. When using a 10× objective, considerably more non-carcinomatous cells were often included in the analysis. However, the ROI functionality of ImmunoRatio can be used to circumvent this problem. The differences in ImmunoRatio analysis results between the tested camera models and repeated staining batches were found to be small (data not shown).

Variation in image brightness and uneven illumination can be accurately corrected by using the blankfield image, captured using the same microscope and camera settings. However, greatly underexposed images (blankfield image mean gray intensity <200) as well as overly overexposed images (blankfield image mean gray intensity >250) may cause false labeling indexes. For accurate nuclei segmentation, the image contrast must be relatively high; the foreground mean gray intensity should be 50 to 80% of the background mean gray intensity. Users can validate their image acquisition settings by using the Camera Adjustment Wizard function of ImmunoRatio.

For the ImmunoRatio web application, it is advantageous to use lossy image file formats (e.g., JPEG) to minimize the data uploaded to the server for analysis. We found that using lossy JPEG compression with quality factors 50 to 100 had no significant effect on the accuracy of ImmunoRatio analysis results (data not shown). This compression level allows a typical 5 megabyte uncompressed image to be compressed into 250 kilobytes (about 20:1 compression ratio), enabling rapid image transfer with almost any network bandwidth. Using very low JPEG quality factors (<50) can cause image distortion and artifacts, making the analysis unreliable.

### Prognostic validation

**Figure 7:**
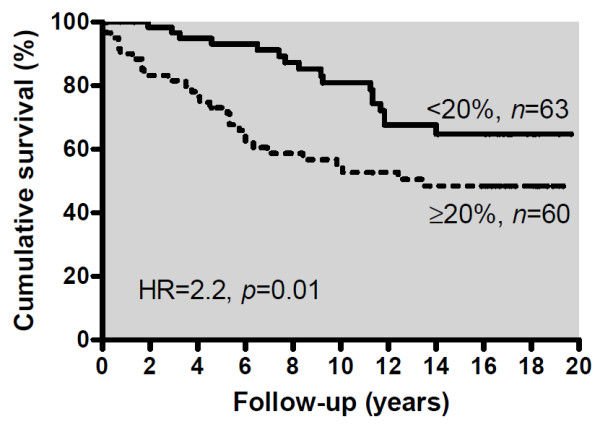
**Breast cancer-specific survival of 123 breast cancer patients according to the Ki-67 labeling index determined with ImmunoRatio**. The cut-off was set at median Ki-67 labeling index (20%). Tumors with a high labeling index were associated with poorer breast cancer-specific survival during the follow up of 20 years (hazard ratio = 2.2, *P *= 0.01).

As Ki-67 is used clinically as a prognostic parameter, we confirmed the accuracy of ImmunoRatio analysis by examining patient survival in a retrospective analysis of 123 breast cancer patients. As expected, based on the literature [5], a strong prognostic correlation was observed (Figure 7). Breast cancer-specific survival of patients with high Ki-67 tumors was significantly shorter than low Ki-67 during 20-year follow-up. Labeling index values of 15%, 20%, and 25% were tested as cut-off. Of those, 20% (the median in this material), gave a hazard ratio (HR) of 2.2 (*P *= 0.01 by log rank test). Cut-off values 15% and 25% yielded similar results (HR = 2.1 and HR = 2.4, respectively, data not shown).

## Discussion

In this study, we described an image analysis application, ImmunoRatio, which is an easy-to-use tool for assessing ER, PR, and Ki-67 labeling indexes in hematoxylin-counterstained tissue sections. ImmunoRatio analysis is based on defining positively stained pixel counts, which, according to our calibration data, correlates very well with cell nuclei enumerated visually. The calibration was performed using a training set of 50 samples and validation using a separate test set of 50 samples representing ER-, PR-, and Ki-67-stained routine breast cancer specimens. The correlation between manual and automated analysis was very high and matched, or exceeded, corresponding results of other similar image analysis software [30,31]. Due to the significant inter-observer variability in visually defined labeling indexes, we recommend that the users calibrate ImmunoRatio with their own labeling index data, as demonstrated in Figure 4 for the calibration training set. Once calibrated, ImmunoRatio can be easily integrated with routine diagnostic work.

Another important aspect of calibration is to determine the optimal Ki-67 cutoff used for prognostic assessment. We tested this with a retrospective analysis of data from 123 primary breast cancer patients followed up for 20 years. The Ki-67 labeling index 20% (the median value in this material) gave a strong prognostic discrimination (HR = 2.2). Although cut-off values 15% and 25% yielded similar prognostication in this patient material, we recommend each laboratory to define their own cut-off value. We recommend using the median value of the Ki-67 labeling index as cut-off. This allows comparisons of different patient materials and provides a reproducible classification of patients according to Ki-67 labeling index.

In addition to accurate calibration, it is clear that for routine use, an image analysis system must accept variation in staining intensity, in microscope setup, and in image acquisition settings. We found up to eight-fold range in primary antibody (Ki-67) dilution to be acceptable for ImmunoRatio. However, when setting up an optimal staining protocol, the users should pay close attention to the hematoxylin counterstaining, which must be bright and clearly separate the nuclei from the background (see example Figure 6). In terms of optical resolution, we recommend using a microscope setup that roughly corresponds to 20× objective lens magnification, 1× phototube, and a 1.5 megapixel camera. Using this setup, a representative result from a typical breast cancer tumor (diameter 1 to 2 cm) can be obtained by averaging at least three images. Variation in image brightness is well-tolerated owing to the blankfield image correction. The Camera Adjustment Wizard function is designed to help the user find the optimal image brightness and contrast settings. A collection of reference images with optimal staining and imaging settings are presented on our website [15].

ImmunoRatio analysis is based on the color deconvolution algorithm [12], which is one of the several existing alternatives for separating the staining components. In addition to color deconvolution, stain separation and nuclei segmentation have been performed using texture analysis [32], cyan-magenta-yellow-black (CMYK) color model [33], hue-saturation-intensity color model [34], CIE 1976 L*u*v (CIELUV) color model [35], pattern recognition [36], cluster analysis [37], and immunofluorescence with Automated QUantitative Analysis (AQUA) [38]. However, the software applications described in the above mentioned studies are mainly for research purposes and they have not been released for public use. Many of the methods may require considerable work if employed in a routine clinical process. The color deconvolution-based approach for separating two stains is straightforward and fast, and is readily usable for images captured with conventional microscope color cameras. If more than two staining components are used or the analysis requires accurate intensity-based quantification, the AQUA method or multispectral imaging would most likely be better alternatives [11].

ImmunoRatio was developed using ImageJ, which is a public domain (i.e., completely free and open source) image analysis software. However, a major obstacle in adopting ImageJ, or any other image analysis software, in clinical laboratories is usually the strict computer security policy. The local system and network rules usually prohibit users to download, install, and/or run external applications. To address these constraints, we released ImmunoRatio as a web application, which provides an easy-to-use web interface, requires no software downloads or installations, and can be used in highly restricted environments.

## Conclusions

To the best of our knowledge, ImmunoRatio is the first ready-to-use web application for analyzing nuclear immunostains (e.g., ER, PR, and Ki-67). We want to point out that ImmunoRatio is meant to be used as a diagnostic aid by personnel trained to score immunostained breast cancer slides. Furthermore, the analysis results should always be interpreted together with the pseudo-colored images and the original sample slides. ImmunoRatio has already been used in the authors' laboratory for more than 1,000 cases and tested by several collaborators. The application is open to free public access on our research group website [15]. Complementary software for analyzing cell membrane staining (e.g., HER-2) is currently being developed.

## Abbreviations

AQUA: automated quantitative analysis; CIELUV: CIE 1976 L*u*v; CMYK: cyan-magenta-yellow-black; DAB: diaminobenzidine; ER: estrogen receptor; HR: hazard ratio; PBS: phosphate-buffered saline; PR: progesterone receptor; ROI: region of interest.

## Competing interests

The authors declare that they have no competing interests.

## Authors' contributions

VJT carried out the software design and implementation, prepared the manuscript, and participated in the study design. SR performed the immunohistochemistry and the software calibration. AV contributed to the software design. MJ supervised the immunohistochemistry and the calibration processes. JI was responsible for the study design and coordination, and helped to draft the manuscript. All authors read and approved the final manuscript.
